# Environmental Groundwater Depth for Groundwater-Dependent Terrestrial Ecosystems in Arid/Semiarid Regions: A Review

**DOI:** 10.3390/ijerph16050763

**Published:** 2019-03-03

**Authors:** Feng Huang, Yude Zhang, Danrong Zhang, Xi Chen

**Affiliations:** 1 College of Hydrology and Water Resources, Hohai University, Nanjing 210098, China; 20030112@hhu.edu.cn; 2 China Water Resources Beifang Investigation, Design and Research Co. Ltd, Tianjing 300222, China; y200427020214@163.com; 3 Institute of Surface-Earth System Science, Tianjin University, Tianjin 300072, China; xichen@hhu.edu.cn

**Keywords:** environmental groundwater depth, definition, methodologies, framework, environmental groundwater regime, arid/semiarid regions

## Abstract

Groundwater in arid/semiarid regions plays crucial roles in providing drinking water supply, supporting irrigated agriculture, and sustaining important native terrestrial ecosystems. Groundwater depth controls water availability to vegetation and is essential for conserving groundwater-dependent terrestrial ecosystems. Environmental groundwater depth can be defined as a mean depth or a range of depths, satisfying the growth of natural vegetation that is not under stress, either due to lack of water or anoxia or soil salinization. Five methodologies have been reported to estimate environmental groundwater depth: the direct ones rely on response functions that relate vegetation condition, e.g., physiological parameters, appearance frequency, community structure, and remotely sensed physical indexes, to changes in groundwater depth; the indirect one estimates environmental groundwater depth based on the threshold of soil moisture content. To fill a knowledge gap of unique recognized methodology, a conceptual framework was proposed, which involves initial estimation (data collection, response assessment, and estimation) and feedback adjustment (implementation and modification). A key component of the framework is to quantify the linkage between ecological conditions and geohydrological features. This review may provide references for groundwater resources management, ecological conservation, and sustainable development in arid/semiarid regions.

## 1. Introduction

Water resources are closely linked to the wellbeing of humankind. Scarcity of water has become a major issue in arid/semiarid regions where scarcity of water is a defining characteristic [1,2]. Groundwater resources are synthetically impacted by global climate and anthropogenic activities, particularly overexploitation of groundwater [3,4]. By increasing temperature, groundwater recharge would be decreased in most places; however, groundwater recharge was increased in some areas due to excessive irrigation return flow [5]. Climate variability at inter-annual timescales related to elevated precipitation and increased runoff associated with El Nino Southern Oscillation (ENSO) caused an increased recharge of aquifers in Arizona, California, and Argentina, as shown by rising groundwater tables [6]. Satellite gravity measurements from the Gravity Recovery and Climate Experiment captured significant depletion of groundwater in many aquifers or regions globally, including Northwest India, High Plains Aquifer, and Central Valley in the USA, North China Plain, Middle East, and Southern Murray-Darling Basin in Australia [7]. To alleviate the growing pressure on water resources, it is essential to implement sustainable water resources management to balance both socio-economic and environmental water requirements [8].

According to the ecological value of groundwater, the groundwater-dependent ecosystems can be generally divided into three types: (a) aquifers and cave ecosystems; (b) springs, wetlands, rivers, estuaries, and nearshore marine ecosystems; and (c) terrestrial vegetation ecosystems [9]. The groundwater-dependent terrestrial ecosystems consist of deep and/or shallow rooted vegetation communities and the fauna that utilizes the habitat formed by the vegetation communities, e.g., reptiles, mammals, and birds [10]. The vegetation in arid/semiarid regions may be completely or partially dependent on groundwater [11]. Alterations in groundwater resources will cause fluctuations of groundwater depth, which is a key environmental factor controlling the availability of water to vegetation [12]. Plant water stress and reduced live biomass is caused by decreasing water tables; however, the converse is not necessarily true for increasing water tables, which can kill flooded roots. In fact, most species cannot tolerate extremely low levels of oxygen [13]. Vegetation may respond to fluctuating availability of groundwater in terms of following processes: leaf-scale photosynthesis, stomatal conductance, canopy conductance, leaf and stem water potential, transpiration, resistance to xylem embolism, growth rate, leaf area index, plant density, crown dieback, mortality, etc. [14]. In arid/semiarid regions, groundwater table fluctuations will not only affect the growth of vegetation but also community structure and ecosystems; while vegetation growth could prevent soil erosion and land desertification [15]. With sustainable utilization of water resources, necessary reserves will not be depleted and groundwater-dependent ecosystems will not be damaged [16].

Hence, a reasonable groundwater depth is necessary to protect the groundwater-dependent terrestrial ecosystems, and a better understanding of environmental groundwater depth is required to enable a determination of the ecological reserves before a water resources plan may be granted or renewed. Zhao and Cheng [17] reviewed some advances in the study of ecohydrological processes in arid zones. They put forward concepts and methods of determining following parameters: critical ecological water requirement, optimal ecological water requirement, and saturated ecological water requirement. Cui and Shao [15] discussed the role of groundwater in arid/semiarid ecosystems, concerning the effect of groundwater depth on growth of plants and degree of soil salinity. They concluded that groundwater depth is a key factor for controlling land desertification and soil salinization in Northwest China. Eamus et al. [11] proposed a functional methodology for determining the groundwater regime needed to maintain the health of groundwater-dependent vegetation. Rohde et al. [18] reviewed global management of groundwater-dependent ecosystems under sustainable groundwater policies. To the best of our knowledge, the importance of a reasonable groundwater depth for sustainable development has been well recognized; however, an updated and synthetic review on how the reasonable groundwater depth can be estimated is necessary and a unique recognized methodology remains a knowledge gap. 

Therefore, this study reviewed pertinent research reported in peer-reviewed international literature that are indexed by Web of Science Core Collection and Chinese Science Citation Database, and aimed to: (a) generalize the definition of environmental groundwater depth; (b) review existing methodologies for estimating environmental groundwater depth; (c) propose a conceptual framework for designing environmental groundwater depth; and (d) discuss future works. The results will be helpful to guide the water resources management and environmental conservation in arid/semiarid regions. 

## 2. Definition of Environmental Groundwater Depth

The importance of groundwater depth for vegetation in arid/semiarid regions has been recognized decades ago, and several concepts have been proposed to define the reasonable ranges of groundwater depth for sustaining the groundwater-dependent terrestrial ecosystems. The concepts mainly include critical groundwater depth [19], ecological groundwater table [20], depth-to-groundwater threshold [14], and critical groundwater level [21]. Although the literal expressions are different, these statements have the same essence with respect to environmental groundwater depth.

**Figure 1:**
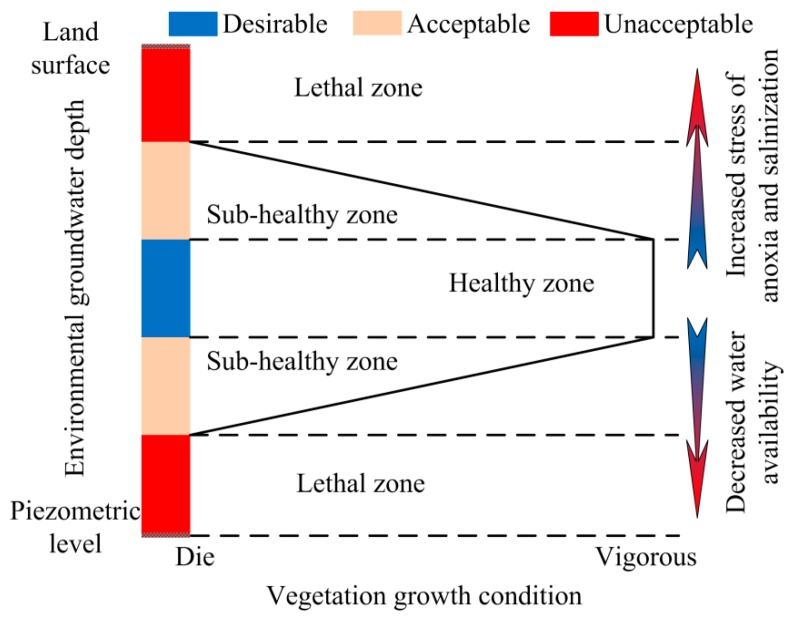
Sketch map for the definition of environmental groundwater depth.

In brief, environmental groundwater depth can be defined as a mean depth or a range of groundwater depths, which satisfy the growth of natural vegetation, not under stress either because of lack of water or anoxia or soil salinization [10,17]. Figure 1 displays a schematic definition of environmental groundwater depth that can be categorized as follows: desirable, acceptable, and unacceptable groundwater depth. Within the desirable range, the capillary fringe is near the vegetation root zone; vegetation grows well and changes in groundwater depth have slight influence on vegetation growth. Beyond the desirable range, a small variation in groundwater depth could significantly affect vegetation; the transition from healthy zone to lethal zone may be linear or non-linear, depending on the specific ecological and hydrological processes and their interactions. When the groundwater level is too low, capillary action cannot lift groundwater upward through the root zone of plants. This could cause soil desiccation, vegetation degradation, and land desertification. However, when the groundwater level is too high, plants could suffer from anoxia; besides that, salts are accumulated on the soil surface, leading to soil salinization through evaporation, and this could cause adverse effects on plant growth. The lower threshold of the acceptable range is critical for water availability, and the upper threshold of acceptable range is critical for soil salinization and vegetation anoxia [15].

## 3. Methodologies for Estimating Environmental Groundwater Depth

Determining environmental groundwater depth is pivotal when integrating environmental considerations into sustainable water resources management in arid/semiarid regions. A reasonable and reliable methodology is necessary for determination. Table 1 lists the methods and their representative applications. It was found that environmental groundwater depths were different for various study areas, mainly because of specific local characteristics of the following entities: climate, hydrology, geology, soil, and vegetation. Furthermore, research results were different even for the same study area, probably because of the differences in data sources and research methodologies.

### 3.1. Fitting Functions between Physiological Parameters and Groundwater Depth

#### 3.1.1. Principles

In arid/semiarid regions, groundwater is significantly important for vegetation growth, which can be characterized by physiological parameters, e.g., leaf stomatal conductance, net photosynthetic rate, shoot water potential, canopy condition, and radial and branch increments. For instance, in the Kangaloon bore-field area of New South Wales in southeastern Australia, structural (leaf-area index, basal area, stem density, tree height, Huber value, and aboveground biomass) and functional (aboveground net primary productivity) attributes of seven woodland sites differing in groundwater depth were examined. Significant differences in structural and functional attributes were observed across sites. The three shallowest sites with 2.4  m, 4.3  m, and 5.5  m groundwater depth had significantly larger aboveground biomass and aboveground net primary productivity than did the four deepest sites where groundwater depth ≥ 9.8  m [30]. The physiological parameters can be related to groundwater depth using scatter plots and regression techniques. Based on the relationship, a function can be fitted and then an abrupt change may be observed which indicates the environmental groundwater depth.

#### 3.1.2. Applications

At the free-flowing Hassayampa River in Arizona (USA), the physiological and growth responses of native riparian trees (*Populus fremontii S. Wats.* and *Salix gooddingii Ball*) to groundwater availability were investigated during dry (1997) and wet (1998) years. In the drier year, the trees experienced considerable water stress, as evidenced by low shoot water potentials, low leaf gas exchange rates, and large amounts of canopy dieback. These parameters were significantly related to groundwater depth. A three-parameter sigmoidal function best described the relationship between canopy dieback and groundwater depth, and suggested an environmental groundwater depth of 2.5–3.0 m, beyond which the canopy dieback of native trees increased rapidly [22].

At the Ejina oases in the lower reaches of the Heihe River, a network of groundwater depth observation sites was monitored, and environmental moisture gradient was reflected by plant physiological characteristics. For salinity control, the threshold of groundwater depth was varied between 0.5–1.5 m. The threshold of groundwater depth for ecological warning was varied between 3.5–4.0 m [23].

### 3.2. Simulating Relationship between Appearance Frequency and Groundwater Depth

#### 3.2.1. Principles

Vegetation tends to grow in its suitable habitat where it can be indicated by a high appearance frequency of the vegetation, and the appearance frequency will decrease with the decreased habitat suitability. Hence, as a key habitat factor of groundwater-dependent terrestrial ecosystems, environmental groundwater depth can be investigated by simulating relationships between appearance frequency and groundwater depth. A high appearance frequency corresponds to an acceptable environmental groundwater depth and vice versa. Gaussian regression is a widely used model to simulate the relationship between species and environment [31]. Because of the complex relations between vegetation and environment in natural vegetation communities, this relationship may not fully comply with the Gaussian regression but a transformed regression, e.g., a lognormal distribution. The ranges of groundwater depth corresponding to the appearance frequency above 20%, above 10%, and below 10% could be estimated as the desirable, acceptable, and unacceptable environmental groundwater depth, respectively. The specific thresholds of appearance frequency are determined by the local targets of environmental conservation.

#### 3.2.2. Applications

The middle and lower reaches of the Tarim River were monitored for two years (2006 and 2007) and data were collected for groundwater, vegetation plots, and soil profiles. The relationships between vegetation and environmental factors were investigated using ecological niche analysis and detrended canonical correspondence analysis. Using ecological suitability theory, data from previous studies were analyzed for several major plant species in the study area. A lognormal distribution model was built to show the relationship between plant growth and groundwater depth. The investigation revealed that plant diversity was highest when groundwater depth was between 2–4 m, followed by groundwater depth of 4–6 m, and then that between 0–2 m. Species diversity decreased dramatically when groundwater depth increased to deeper than 6 m. For major plant growth, the optimum groundwater depth was in the range of 2–4 m; the threshold of groundwater depth was about 6 m. To restore the vegetation and ecosystem of the lower reaches of Tarim River, the depth to groundwater table must be kept at a minimum value of 6 m. The groundwater depth must be maintained at 2–4 m in the vicinity of water way and at 4–6 m for the remaining section of this arid area [24].

### 3.3. Identifying Responses of Vegetation Community Structure to Alterations in Groundwater Depth

#### 3.3.1. Principles

The distribution of vegetation in arid/semiarid regions is closely related to groundwater depth. A good correlation exists between groundwater depth and supergene ecological type [32]. For example, in the Cele oasis of China, as groundwater depth varies from deep to shallow, vegetation communities transform with following sequences of *Alhagi* communities, *Tamarix* spp. communities, *Populus* communities, *Phragmites* communities, and *Sophora* communities [33]. Different species have different capacities to adapt and respond to alterations in groundwater availability, which progressively affect the vegetation growth, reproduction, mortality, and community structure. Although vegetation has evolved complex physiological and biochemical adaptations to water deficit or inundation stress, the adaptations may become inadequate and lead to a shift in the vegetation community structure, if the stress is so extreme that exceeds the tolerance of vegetation. When groundwater table keeps chronically declining, the vegetation community may degenerate from trees/shrubs/herbs to sparse trees, and even just a barren. Therefore, by identifying the responses of vegetation community structure to changes in groundwater depth, the threshold of groundwater depth corresponding to prominent ecosystem degeneration can be set as an environmental management target.

#### 3.3.2. Applications

Based on in situ observations of groundwater depth and the length, width, and fresh weight of 50 leaves of selected plants in July 2010, the response of vegetation communities was investigated by altering groundwater depth. The results indicated that herbs exhibited degradation when groundwater depth was 4–6 m, whereas trees did not exhibit degradation until groundwater depth exceeded 6 m in the lower reaches of the Tarim River. The mixed distribution of trees/shrubs/herbs was conspicuous in areas where groundwater depth was in the range 2–4 m; the distribution of trees/shrubs was conspicuous in areas where the groundwater depth was in the range 4–8 m. In areas where the groundwater depth was greater than 8 m, the simple structure of degraded *Populus euphratica*/*Tamarix chinensis* dominated the region [25].

In the Ejina oases at the lower Heihe River, the relationship between vegetation and groundwater depth was investigated in June 2000 by combining remote sensing with in situ groundwater measurements. The results indicated that groundwater depth suitable for vegetation in this region was in the range 2–5 m, depending on species composition. Hardly any vegetation occurred when groundwater depth was below 5.5 m, because rooting depth of present species was limited. Therefore, they could not maintain adequate water supplies to their canopies [26].

### 3.4. Investigating the Relationship between Remotely Sensed Physical Indexes of Vegetation and Groundwater Depth

#### 3.4.1. Principles

Remote sensing provides a robust and spatially explicit means to assess land cover and its temporal changes. Vegetation coverage, which is highly correlated with groundwater availability in arid/semiarid regions, tends to be larger when the groundwater availability is better. The remotely sensed indexes of vegetation include normalized difference vegetation index (NDVI), enhanced vegetation index (EVI), perpendicular vegetation index (PVI), soil adjusted vegetation index (SAVI), and transformed soil adjusted vegetation index (TSAVI) [34,35,36,37,38]. The relationships between the remotely sensed indexes and groundwater depth may be linear, curvilinear, or step-wise related; they are most likely case specific because of localized variations in vegetation communities, hydrogeological conditions and climatic regimes. Using these relationships, environmental groundwater depth can be estimated through a breakpoint, if it exists in a step-wise relationship, or based on the management target of environmental conservation.

#### 3.4.2. Applications

In the Owens Valley, California, USA, cloud-free Landsat Thematic Mapper and Enhanced Thematic Mapper plus satellite data provided the information of vegetation coverage. Based on the correlation analysis of vegetation coverage and groundwater depth, the threshold of groundwater depth was estimated to be 2.5 m; it represented the average plant rooting depth and prevented adverse effects on vegetation cover and plant assemblages [27].

In the hyper-arid Atacama Desert of northern Chile, Landsat images were complemented with a digital inventory and estimation of the green canopy fraction of all trees. Novel remote sensing drought stress indicators were defined based on the NDVI. A spatio-temporal assessment of the water condition of *Tamarugo* trees indicated that the paraheliotropic leaf movement was limited when groundwater depth was greater than 12 m. This caused dehydration and foliage loss. *Tamarugos* at 12–16 m of groundwater depth suffered moderate drought stress while severe drought stress was incurred by 16–20 m of groundwater depth. Therefore, 20 m of groundwater depth was considered as a critical threshold for the survival of *Tamarugo* trees [28].

### 3.5. Estimation Bbased on the Threshold of Soil Moisture Content

#### 3.5.1. Principles

Soil moisture is one of the most important components of soil and a basic condition on which vegetation relies for survival. In arid/semiarid regions, especially during dry periods with sparse precipitation, groundwater has an essential effect on soil moisture content which influences local ecological stability. Hence, based on the threshold of soil moisture content that vegetation can endure and the relationship between soil moisture content and groundwater depth, environmental groundwater depth can be estimated. This method is an indirect method, while the above four methods are direct ones. 

#### 3.5.2. Applications

In the lower reaches of the Tarim River (China), the variability of soil moisture content and the relationship between soil moisture content, groundwater depth, and vegetation were analyzed using the methods of coefficient of variation, Pearson correlation, and regression, respectively. The findings suggested that plant coverage and species diversity improved significantly with increasing soil moisture content in the soil layer of 100–260 cm. Plant coverage and species diversity approached maximum values, and 92.3% of major plant species were able to grow when soil moisture content was greater than 10%. To restore the ecosystem of desert riparian forest along the lower reaches of the Tarim River, groundwater depth had to be maintained at <4 m in the vicinity of watercourse and at about 4 m for the remaining section of this arid region [29].

## 4. A Conceptual Framework for Designing Environmental Groundwater Depth

**Figure 2:**
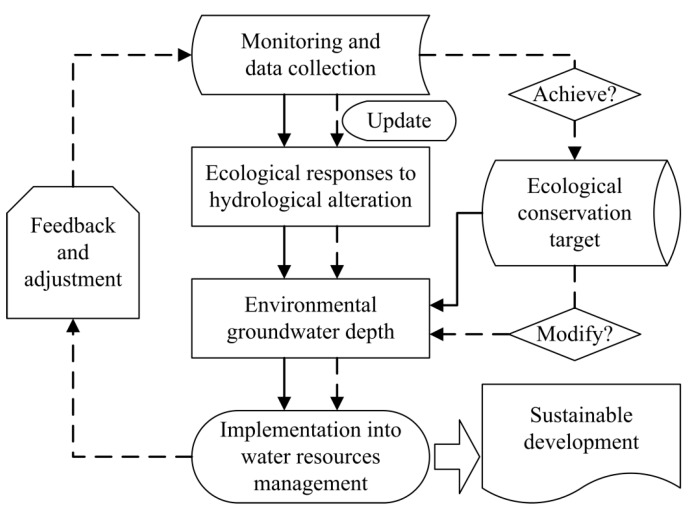
A conceptual framework for designing environmental groundwater depth. Note: solid lines indicate the preliminary processes and dashed lines denote the follow-up feedback processes.

Based on the review of existing methodologies for estimating environmental groundwater depth, a conceptual framework was proposed to guide the practice of water resources management and achieve sustainable development in arid/semiarid regions. The appraisal processes mainly includes five steps (Figure 2), which can be categorized into two processes of preliminary estimation and adaptive adjustment. 

*Step 1: Monitoring and Data Collection*

A long-term program of monitoring both hydrological processes and the condition of groundwater-dependent terrestrial ecosystems is the basis for the sustainable water resources management. The data can be collected through in situ field surveys, remote sensing technologies, and the combination of the two methods.

Groundwater levels are highly dynamic in nature and measurement of groundwater levels requires systematic networks of observation wells; an effective groundwater monitoring network design is necessary to provide more precise qualitative and quantitative information on complex aquifer systems for optimal groundwater management [39]. The commonly applied methodologies for designing groundwater monitoring networks are principal component analysis, simulated annealing, entropy theory, and geostatistics [40,41,42,43,44]. 

The responses to changes in groundwater depth could be quantified at all ecological levels, including individual, population, and community levels; an individual species response has implications for population response which further affects community composition or structure. When deciding which traits of groundwater-dependent terrestrial ecosystems should be measured, there are several criteria required to be considered: having a defined relationship with groundwater depth, characterizing risk to the environment, cost-effective and practical, having early warning capabilities, and reflecting the “lag” effects [11]. Eamus et al. [11] have discussed which features of vegetation can be measured to monitor ecosystem function and provided a range of techniques that are available to measure the aspects of ecosystem function. 

*Step 2: Assessing Ecological Responses to Hydrological Alteration*

The collected data are used to establish how groundwater-dependent terrestrial ecosystems are impacted by groundwater fluctuations; the relationships between ecological traits and groundwater depth could be analyzed by taking groundwater depth as a driving factor of ecological responses. It is a key step to investigate the ecological responses to altered groundwater depth and simulate the response functions, which could depend a lot on site conditions that mainly include climate, topography, soil, and vegetation species [13,45,46]. Such relationships may provide scientific reference to estimate environmental groundwater depth for other regions with similar site conditions, where long-term monitoring data are not available. 

The responses have been examined extensively at leaf, tree, canopy, population, and community scales; and the response functions for individual vegetation traits are readily apparent [14]. When integrating multiple-scale responses and developing an ecosystem-scale response function for groundwater depth, a practical approach is to normalize the responses (0 to 1) such that a response of 1 indicates no effect of differences in groundwater depth and 0.5 indicates a 50% decline/increase in the maximal/minimum value of a particular trait [47]. When applying statistical analysis to identify associations between ecosystem condition and groundwater depth, special attention must be paid to possible time lags in ecological responses to groundwater table fluctuations. The losses of riparian trees may show lags of years to decades before becoming evident because it requires exceptional droughts before trees pass stress thresholds and die [48].

*Step 3: Estimating Environmental Groundwater Depth*

Once the linkage between the processes of ecology and geohydrology is established, it becomes exercisable to determine the environmental groundwater depth to maintain a particular condition of the ecosystems. The estimation takes two forms:

(i) Detecting a significant “jump point”, if it exists, to identify the threshold of groundwater depth, exceeding which noticeable ecological degradation occurs. The statistical methods for identifying thresholds, e.g., Bayesian inference approach combined with the Markov Chain Monte Carlo sampling method for a simplified vegetation dynamic model, and Threshold Indicator Taxa Analysis [49,50], are applicable. Andersen et al. [51] have provided a review on approaches and software for the identification of ecological thresholds and regime shifts. 

(ii) Analyzing environmental groundwater depth based on the graph of relation between ecological indexes and groundwater depth and a pre-set goal of ecological conservation. This method is applicable no matter whether the “jump point” exists in the graph of relation or not. The target of ecological conservation can be decided by basin managers, who can consult the stakeholders in the catchment during the decision-making process and take into account the relative importance of the groundwater-dependent terrestrial ecosystems. 

*Step 4: Implementation into Water Resources Management*

Environmental groundwater depth, which is a target of water resources management, limits the amount of water that can be withdrawn from the aquifer and informs the manager how much water should remain. After the preliminary estimation, the environmental groundwater depth can be applied in the water resources management. The application provides feedback for adaptive management.

*Step 5: Feedback and Adjustment*

The initial estimation is a part of the multi-step conceptual framework that concludes with final determination of environmental groundwater depth. When the preliminarily estimated environmental groundwater depth is applied, the monitoring strategy will be implemented continuously to support the adaptive management. The updated data will contribute to refine or further confirm the ecological responses to hydrological alteration. The ecological monitoring informs whether the ecological conservation target has been achieved. If the initial environmental groundwater depth fails to sustain the pre-set ecological condition or a new ecological conservation target is set, the environmental groundwater depth needs to be adjusted using the updated graph of linkage between ecology and geohydrology. The re-estimated environmental groundwater depth will also be applied in water resources management. The feedback and adjustment will continue until a sustainable development is achieved.

## 5. Discussion on Environmental Groundwater Regime

Groundwater level usually experiences diurnal, seasonal, and inter-annual fluctuations, which should be incorporated into the design of environmental groundwater depth. However, the published papers mainly concerned on the static thresholds of groundwater depth (Table 1) and have not taken the natural groundwater regime into account yet, leaving a knowledge gap in dynamic environmental groundwater depth. 

The natural groundwater regime includes magnitude, timing, duration, change rate, and frequency of the geohydrological event, similar with the natural flow regime in a river [11,52]. The magnitude is the value of groundwater depth, which varies with months or seasons. The timing characterizes when a specific geohydrological event happens. For example, when do the highest and lowest groundwater levels occur in a hydrological year? The duration is total days that a geohydrological event continues. The vegetation may have some adaptive strategies to endure a drought with a short-term duration; however, it may not survive due to a prolonged drought without groundwater availability. The change rate quantifies how fast groundwater level rises or declines. Some vegetation is capable of adjusting to the drop of groundwater level because of high root growth rate; the root system will be adapted to keep in contact with the falling groundwater table [10,53,54,55]. However, if the falling rate of groundwater level is much too large, the root system will have no time to adjust and may result in mortality of the vegetation. The frequency indicates how many times a specific geohydrological event, e.g., drought and inundation, happens in a year. 

The groundwater-dependent terrestrial ecosystems have adapted to the natural groundwater regime, which should not be fundamentally destroyed when exploiting the groundwater resources. An environmental groundwater regime, which is vividly analogous with the natural one, can better contribute to ecological conservation. Hence, more attention is suggested to be paid to the environmental groundwater regime in future works. The framework for designing the environmental groundwater regime is similar with the framework guiding the estimation of environmental groundwater depth. An improvement is embodied in an enhanced analysis of the linkage between the processes of ecology and geohydrology. In the graph of ecological responses to groundwater alterations, the factors that cause ecological changes include not only groundwater depth, which is the magnitude of groundwater regime, but also timing, duration, change rate, and frequency of groundwater fluctuations.

## 6. Conclusions

Environmental groundwater depth which is an important hydrogeological parameter controlling the water availability to vegetation is crucial for ecological conservation in arid/semiarid regions. Its importance has been well recognized, and the peer-reviewed publications have reported five methods for quantifying it, among which are four direct methods and one indirect method. The four direct methods are commonly based on the response of vegetation traits to changes in groundwater depth; the difference is the applied vegetation traits, which include physiological parameters, appearance frequency, community structure, and remotely sensed physical indexes. The indirect method firstly quantifies the threshold of soil moisture content for vegetation growth; then it analyzes environmental groundwater depth based on the relationship between soil moisture content and groundwater depth. The direct methods are lower cost than indirect ones and could be widely applied; however, they only provide an initial estimation and a feedback adjustment is missing. Therefore, a conceptual framework was proposed to design environmental groundwater depth. The existing methodologies could be incorporated into this framework for the preliminary estimation, the result of which will be applied into water resources management and the initial environmental groundwater depth will be adjusted using the updated eco-hydrological response curve, if the ecological conservation goal is not actually achieved. This framework would be practical for the sustainable development of arid/semiarid regions. 

The environmental groundwater depth mainly concerns the magnitude of depth to groundwater table, ignoring the natural variations of geohydrological processes. More attention was suggested to be paid to the environmental groundwater regime, the five components of which involve magnitude, timing, duration, change rate, and frequency. It is necessary to further develop quantitative information on ecological responses to alterations in groundwater regime. The adaptive assessment is also applicable to design the environmental groundwater regime. 

The present study focused on peer-reviewed publications; however, restrained by data availability, this review could not consider unpublished materials, e.g., research reports and academic presentations. Hence, a deficiency is that the synthesis of methodologies for estimating environmental groundwater depth might possibly be incomplete.

## Figures and Tables

**Table 1:** Typical achievements of environmental groundwater depth.

Methodology	Study Area	Environmental Groundwater Depth			Reference
		Desirable	Acceptable	Unacceptable	
Fitting functions between physiological parameters and groundwater depth	The Hassayampa River, Arizona, USA			>2.5–3.0 m	[22]
	The Ejina oases, the lower Heihe River, China			<0.5–1.5 m >3.5–4.0 m	[23]
Simulating relationship between appearance frequency and groundwater depth	The middle and lower Tarim River, China	2–4 m	4–6 m	>6 m	[24]
Identifying responses of vegetation community structure to alterations in groundwater depth	The lower Tarim River, China	2–4 m	4–8 m	>8 m	[25]
	The Ejina oases, the lower Heihe River, China		2–5 m	>5.5 m	[26]
Investigating the relationship between remotely sensed physical indexes of vegetation and groundwater depth	The Owens Valley, California, USA			>2.5 m	[27]
	The Atacama Desert, northern Chile			>20 m	[28]
Estimation based the threshold of soil moisture content	The lower Tarim River, China	<4 m			[29]
